# Quantitative analysis of early apparent diffusion coefficient values from MRIs for predicting neurological prognosis in survivors of out-of-hospital cardiac arrest: an observational study

**DOI:** 10.1186/s13054-023-04696-z

**Published:** 2023-10-25

**Authors:** Jung A. Yoon, Changshin Kang, Jung Soo Park, Yeonho You, Jin Hong Min, Yong Nam In, Wonjoon Jeong, Hong Joon Ahn, In Ho Lee, Hye Seon Jeong, Byung Kook Lee, Jae Kwang Lee

**Affiliations:** 1 Department of Emergency Medicine, Chungnam National University Hospital, Daejoen, Republic of Korea; 2 Department of Emergency Medicine, College of Medicine, Chungnam National University, 266 Munwha-ro, Jung-gu, Daejeon, 35015 Republic of Korea; 3 Department of Emergency Medicine, Sejong Chungnam National University Hospital, Daejoen, Republic of Korea; 4 Department of Radiology, College of Medicine, Chungnam National University, 266, Munhwa-ro, Jung-gu, Daejeon, Republic of Korea; 5 Department of Neurology, Chungnam National University Hospital, Daejeon, Republic of Korea; 6 Department of Emergency Medicine, Chonnam National University Medical School, Chonnam National University Hospital, Gwangju, Republic of Korea; 7 Department of Emergency Medicine, College of Medicine, Konyang University Hospital, Daejeon, Republic of Korea

**Keywords:** Cardiac arrest, Prognosis, Brain, Computed tomography, Magnetic resonance imaging

## Abstract

**Background:**

This study aimed to quantitatively analyse ultra-early brain diffusion-weighted magnetic resonance imaging (DW-MRI) findings to determine the apparent diffusion coefficient (ADC) threshold associated with neurological outcomes in comatose survivors of out-of-hospital cardiac arrest (OHCA).

**Methods:**

This retrospective study included adult survivors of comatose OHCA who underwent DW-MRI imaging scans using a 3-T MRI scanner within 6 h of the return of spontaneous circulation (ROSC). We investigated the association between neurological outcomes and ADC values obtained through voxel-based analysis on DW-MRI. Additionally, we constructed multivariable logistic regression models with pupillary light reflex (PLR), serum neuron-specific enolase (NSE), and ADC values as independent variables to predict poor neurological outcomes. The primary outcome was poor neurological outcome 6 months after ROSC, determined by the Cerebral Performance Category 3–5.

**Results:**

Overall, 131 patients (26% female) were analysed, of whom 74 (57%) showed poor neurological outcomes. The group with a poor neurological outcome had lower mean whole brain ADC values (739.1 vs. 787.1 × 10^–6^ mm/s) and higher percentages of voxels with ADC below threshold in all ranges (250–1150) (all *P* < 0.001). The mean whole brain ADC values (area under the receiver operating characteristic curve [AUC] 0.83) and the percentage of voxels with ADC below 600 (AUC 0.81) had the highest sensitivity of 51% (95% confidence interval [CI] 39.4–63.1; cut-off value ≤ 739.2 × 10^−6^ mm^2^/s and > 17.2%, respectively) when the false positive rate (FPR) was 0%. In the multivariable model, which also included PLR, NSE, and mean whole brain ADC values, poor neurological outcome was predicted with the highest accuracy (AUC 0.91; 51% sensitivity). This model showed more accurate prediction and sensitivity at an FPR of 0% than did the combination of PLR and NSE (AUC 0.86; 30% sensitivity; *P* = 0.03).

**Conclusions:**

In this cohort study, early voxel-based quantitative ADC analysis after ROSC was associated with poor neurological outcomes 6 months after cardiac arrest. The mean whole brain ADC value demonstrated the highest sensitivity when the FPR was 0%, and including it in the multivariable model improved the prediction of poor neurological outcomes.

**Supplementary Information:**

The online version contains supplementary material available at 10.1186/s13054-023-04696-z.

## Background

Despite improvements in post-cardiac arrest care, such as target temperature management (TTM), many survivors of cardiac arrest remain comatose and subsequently die of hypoxic-ischaemic brain injury (HIBI) [1–6]. HIBI measurement in the early phase after the return of spontaneous circulation (ROSC) is important to support attending physicians in explaining and counselling the patient’s family about the patient’s condition and aiding in decision-making [7–10]. As it can be a burden on patients’ families and public healthcare systems in terms of cost and use of medical resources, patients who could potentially have a good neurological prognosis might have been hindered from receiving appropriate treatment opportunities in the past [11].

Current international guidelines for post-cardiac arrest care recommend predicting neurological outcomes using a multimodal approach at least 72 h after ROSC [5]. Among the various predictive tools for neurological prognostication, neuroimaging has the advantage of being unaffected by the sedatives and paralytics used in post-cardiac arrest care [12–16]. In the early stages, only brain computed tomography (CT) is recommended as a predictive tool; however, the grey/white matter ratio (GWR) on CT within 2 h of ROSC underestimates HIBI, limiting its use as an early prognostic tool [12].

In contrast, magnetic resonance imaging (MRI) is useful for identifying cytotoxic oedema and shows a higher sensitivity than CT for identifying and quantifying HIBI [16–19]. Recently, we reported that the presence of high-signal intensity (“restricted diffusion”) on ultra-early (within 6 h after ROSC) diffusion-weighted MRI (DW-MRI) was associated with poor neurological outcomes 6 months after ROSC [7]. However, there are still inter-observer differences and analysis of the extent of HIBI using a manual scoring system presents limitations [18, 19]. The apparent diffusion coefficient (ADC) is a measure of the magnitude of restricted diffusion within a voxel of brain tissue. Lower ADC values indicate more restricted diffusion [1, 2, 5, 13, 20], and quantitative analysis using ADC values calculated semi-automatically with software programs, such as the FMRIB Software Library (FSL), provides accurate information on the severity and extent of HIBI. In adults, it is known that an ADC below 650 × 10^−6^ mm^2^/s in more than 10% of brain tissue obtained through MRI between 4 and 7 d after cardiac arrest can predict a poor neurological outcome with high specificity [20–23]. However, to the best of our knowledge, the appropriate ADC value and threshold for predicting poor neurological outcomes using an MRI obtained within 6 h after the ROSC have not yet been reported.

Therefore, in this study, we aimed to investigate the association between ADC values based on voxel quantification in DW-MRI and poor neurological outcomes at 6 months post-ROSC. We also sought to identify the optimal MRI-based ADC metrics (ADC value and thresholds) and compare their predictive performance with CT-based GWR values. Additionally, we aimed to examine whether the inclusion of ADC metrics, in conjunction with neurological examination features and biomarkers, in a multivariable prediction model, could improve the accuracy of prognostic prediction for ROSC outcomes at 6 months.

## Methods

### Study design and population

In this retrospective observational study, we used prospectively collected data from adult comatose out-of-hospital cardiac arrest (OHCA) survivors treated with TTM at Chungnam National University Hospital (CNUH) in Daejeon, Korea, between May 2019 and December 2022. A part of this study’s data—collected between May 2019 and February 2022 (106 patients)—overlapped with that of a previously published study on the association between ultra-early diffusion-weighted image (DWI) and neurological outcomes [7]. This study was approved by the Institutional Review Board of CNUH (CNUH-2023-03-089). Written informed consent was obtained from all patients and/or their legal guardians(s) and registered in a database.

The inclusion criteria were adult patients (≥ 18 years) who had survived OHCA, were treated with TTM, and underwent MRI scans using a 3 T scanner within 6 h of ROSC. The exclusion criteria were: patients whose cardiac arrest was caused by trauma, those with evidence of severe brain atrophy or a sequelae of a previous injury (such as stroke, tumour, and intracerebral haemorrhage) on MRI, those with poor neurological status before the OHCA (i.e. coma or vegetative state), those who received extracorporeal membrane oxygenation (ECMO), and those whose MRI scan time exceeded 6 h after ROSC.

### TTM protocol

All the patients included in this study underwent TTM. A target temperature of 33 or 36 °C was maintained for 24 h using an Arctic Sun® (Arctic Sun® 5000, BD, Franklin Lakes, NJ, USA) feedback-controlled surface cooling device. Our institution had previously set the target temperature at 33 °C before March 2022. However, after that, the attending physician chose between 33 and 36 °C depending on the patient’s haemodynamic status or cardiac arrest characteristics (cardiac vs. non-cardiac). Upon completion of the TTM maintenance period, the patients were rewarmed to 37 °C at a rate of 0.25 °C/h. All patients received sedatives and neuromuscular blocking agents during the TTM. They also received standard intensive care according to our institutional intensive care unit protocol based on the international guidelines for post-cardiac arrest care [5]. Until February 2018, withdrawal of life-sustaining treatment (WLST) was not permitted in South Korea unless a patient had been pronounced brain dead; even after that, WLST was rarely performed during TTM.

### Data collection

We extracted the following data from our prospective registry: age, sex, Charlson Comorbidity Index (CCI), preexisting illness, aetiology of cardiac arrest (cardiac vs. non-cardiac), witnessed arrest, bystander cardiopulmonary resuscitation (CPR), first monitored rhythm (shockable vs. non-shockable), time from collapse to CPR (no-flow time), time from CPR to ROSC (low-flow time), time to perform MRI and/or CT scans from ROSC, serum lactic acid level after ROSC, and target temperature (33 vs. 36 °C). Furthermore, we extracted data on predictors measured within 6 h of ROSC: serum neuron-specific enolase (NSE) levels, the presence or absence of the pupillary light reflex (PLR), and the GWR on CT.

### Outcome

We evaluated the neurological outcomes of the patients 6 months after ROSC using the Glasgow–Pittsburgh cerebral performance category (CPC) scale. The CPC score classifies patients into five categories: CPC 1 (good performance), CPC 2 (moderate disability), CPC 3 (severe disability), CPC 4 (vegetative state), and CPC 5 (brain death or death). The neurological outcome was dichotomised into good (CPC 1–2) and poor (CPC 3–5). It was conducted either through face-to-face or telephone interviews, and the primary outcome was poor neurological outcome.

### MRI, image processing and analysis

**Fig. 1:**
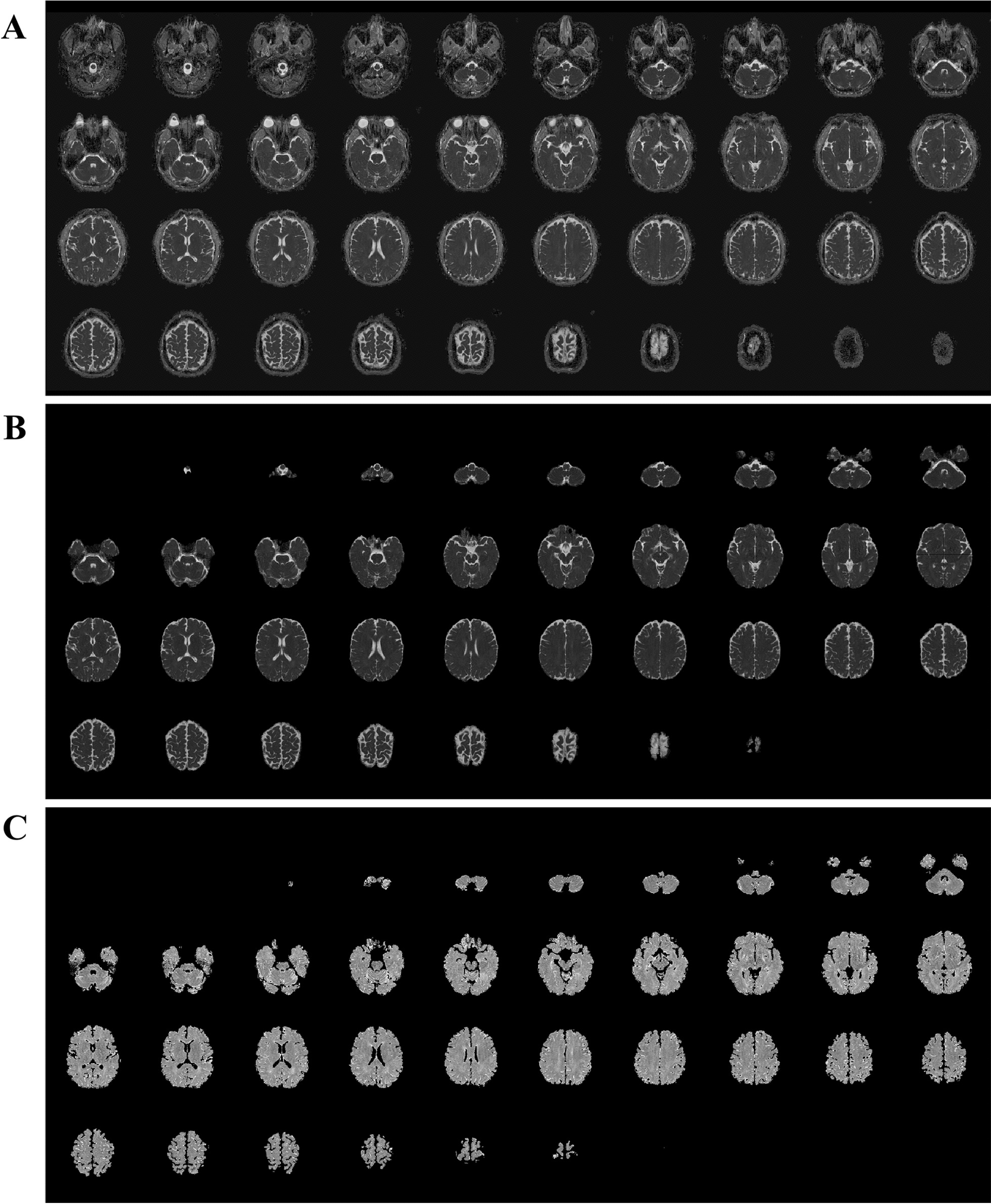
Semi-automated artefact removal algorithm using the FMRIB Software Library. **A** Read the apparent diffusion coefficient MRI image in Digital Imaging and Communication in Medicine format. **B** Remove the cranium, optical structures, and extracranial soft tissues. The brain parenchyma and CSF are extracted. **C** Remove the space of cerebrospinal fluid (black colour). Brain parenchyma is extracted

Our TTM protocol recommends, but does not require, obtaining brain MRI scans within 6 h after ROSC. The MRI was performed using a 3 T scanner (Intera Achieva; Philips Healthcare, Best, Netherlands), which included DWI, ADC maps, and T2-weighted imaging. DWI with b values of 0, 1000, and 3000 is conventionally performed in the axial plane using three orthogonal directions of the diffusion-sensitising gradients combined into isotropic images. In this study, we used DW-MRI with a b value of 1000. For voxel-based quantitative analysis of ADC, FSL software (Release 5.0 ^©^ 2012, The University of Oxford) was used to semi-automatically remove the cranium, optic structures, and extracranial soft tissues. Images were retrieved in Digital Imaging and Communication in Medicine format from picture archiving and communication system servers at the hospital and were converted to the Neuroimaging Informatics Technology Initiative format using MRIcron software (http://www.nitrc.org/ projects/mricron) (Fig. 1). We then calculated the percentage of voxels below different ADC thresholds and the mean whole brain ADC values. To reduce errors caused by artefacts, noise, and fluid content, voxels with ADC values greater than 2000 × 10^−6^ mm^2^/s were extracted from the analysis [8]. The ADC thresholds ranged from 200 to 1200 × 10^−6^ mm^2^/s with a step of 50, and the percentage of voxel with ADC values below the threshold (ADC-PercentValue threshold) was calculated for each ADC threshold up to 1150 × 10^−6^ mm^2^/s [21]. To calculate the ADC-PercentValue threshold, the total sum of the voxels from 200 to the specified interval was calculated, and then, the value was divided by the total sum of the voxels within the range of 200–2000 [21]. The mean whole brain ADC value was defined as the average ADC value of the entire brain volume. Technically inadequate images were excluded from the analysis and all other images were evaluated by an emergency medicine specialist (J.H.M.) with over 10 years of clinical experience, who was blinded to the patient's clinical course and outcomes.

The percentage of voxels with ADC value below threshold was calculated as.

$${\text{ADC}} - {\text{Percent}}\,{\text{Value}}\,{\text{threshold }}\left( \% \right) = \frac{{{\text{Sum}}\,{\text{of}}\,{\text{the}}\,{\text{voxels}}\,{\text{with}}\,{\text{ADC}}\,{\text{value}}\begin{array}{*{20}c} {{\text{threshold}}} \\ {200} \\ \end{array} }}{{{\text{Sum}}\,{\text{of}}\,{\text{the}}\,{\text{voxels}}\,{\text{with}}\,{\text{ADC}}\,{\text{value}}\begin{array}{*{20}c} {2000} \\ {200} \\ \end{array} }} \times 100$$

### Measurement of GWR using brain CT scans

Our TTM protocol recommends obtaining brain CT scans immediately after ROSC. Non-contrast CT scans were obtained in 5-mm slices using a 64-channel system (Somatom Sensation 64, Siemens Healthineers, Munich, Germany). A board-certified neuroradiologist (I.H.L) who was blinded to clinical outcomes, measured the Hounsfield unit of the putamen (P), the caudate nucleus (CN), the posterior limb of the internal capsule (PIC), and the corpus callosum (CC) and calculated the GWR ([P + CN] / [PIC + CC]). The regions were manually measured in circular shapes, approximately 9–12 mm^2^ in size at the level of the basal ganglia including P, CN, PIC, and CC.

### Measurement of PLR and NSE

We defined the absence of PLR as the absence of bilateral PLR 6 h after ROSC. At our institution, PLR was measured using a penlight every hour after ROSC; neuromuscular blockers were not used, but sedatives were occasionally used. Serum NSE levels were measured using the values obtained within 6 h of ROSC. Our institution’s TTM protocol involves measuring serum NSE levels as soon as possible after ROSC, as well as at 24, 48, and 72 h. All samples were analysed in the same laboratory, GC Labs (Yongin, Gyeonggi-do, Korea), and aliquots heavily contaminated with blood or haemolysed blood were discarded. NSE levels were measured using an electrochemiluminescence immunoassay kit (COBAS1 e801; Roche Diagnostics, Basel, Switzerland) [24].

### Statistical analysis

Categorical variables are presented as numbers with percentiles, and continuous variables are presented as means with standard deviations or medians with interquartile ranges (IQRs), according to the normality test. Categorical variables were compared between groups using the *χ*^2^ tests with continuity correction in the 2 × 2 tables or Fisher’s exact test. Continuous variables were compared using Student's t test or Mann–Whitney U test, as appropriate. Receiver operating characteristic (ROC) curves were constructed to evaluate the prognostic performance of single predictors and their combination models to predict poor neurological outcomes at 6 months. An optimal cut-off value was established using 100% specificity (i.e. a false positive rate [FPR] of 0). Areas under the ROC curves (AUC) were compared using the DeLong test [25]. An AUC of 0.50–0.69, 0.70–0.79, 0.80–0.89, and 0.90–1.00 represented poor, fair, good, and excellent prognostic performance, respectively [26]. We performed a logistic regression analysis to identify the independent risk factors for poor neurological outcomes among various neuroimaging parameters (mean whole brain ADC value and ADC-PercentValue thresholds) performed within 6 h after the ROSC. All variables with *P* < 0.1 in univariable analyses were included in the multivariable logistic regression model. A backward-selection method was used to develop the final model. Logistic regression analysis results are reported as odds ratios (ORs) with 95% confidence intervals (95% CIs). Furthermore, we selected the mean whole brain ADC value and ADC-PercentValue thresholds with high accuracy and sensitivity at a 0% FPR and added them to the baseline prediction model (PLR and serum NSE levels) using multivariable logistic regression analysis. We performed statistical analysis using SPSS version 26.0 (IBM Corp., Armonk, NY, USA) and the MedCalc program version 15.2.2 (MedCalc Software, Mariakerke, Belgium). The significance level was set at *P* < 0.05.

## Results

### Patient characteristics

**Fig. 2:**
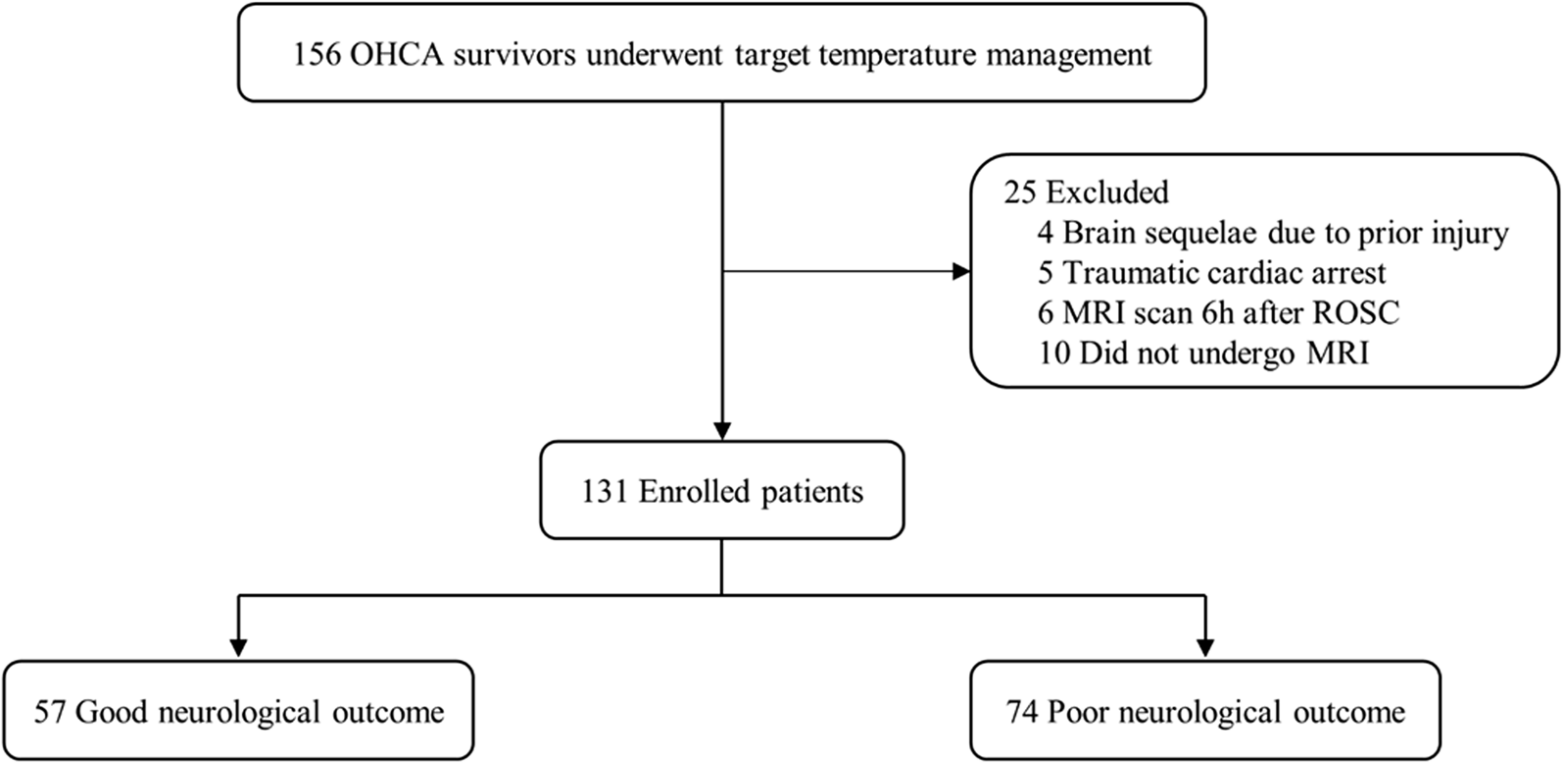
Flow diagram of included study patients. OHCA, out-of-hospital cardiac arrest; MRI, magnetic resonance imaging; ROSC, return of spontaneous circulation

Of the 156 comatose survivors of OHCA treated with TTM during the study period: four had evidence of brain sequelae due to prior injury, five had a CA due to trauma, six underwent an MRI scan 6 h after ROSC, and 10 did not undergo MRI scans. Therefore, 131 patients were included in the study. Notably, 6 months after ROSC, 57 (44%) and 74 (56%) patients were assigned to the good and poor neurological outcome groups, respectively (Fig. 2). The demographic and OHCA characteristics stratified according to neurological outcomes at 6 months are shown in Table 1. The median time from ROSC to CT and DW-MRI was 1.2 (1.1–1.3) and 2.9 (2.0–3.9) h, respectively. The good neurological outcome group had a higher proportion of patients with witnessed arrest, presence of bystander CPR, shockable rhythms, cardiac aetiology, and shorter no-flow and low-flow times. However, there were no significant differences between the two groups regarding age, sex, CCI, and time from ROSC to CT and MRI scan. In addition, the group with good neurological outcomes had higher GWR values and presence of PLR but lower serum NSE levels than in the poor outcome group. Among the included patients, 11 (8.3%) died during TTM (4 [36.4%] due to cerebral injury and 7 [63.6%] due to circulatory failure), and 50 (38.2%) died after TTM (11 [22.0%] due to organ donation, 6 [12.0%] due to WLST, 12 [24.0%] due to unknown causes, and 21 [42.0%] due to cerebral injury). The median time between ROSC and death for patients who died during the TTM period was 2 days (IQR, 2–3), while it was 11.5 days (IQR, 6–31) for those who died after the completion of TTM.

**Table 1:** Baseline demographic data and cardiac arrest characteristics

Characteristics	Overall cohort, n = 131	Good neurological outcome, n = 57	Poor neurological outcome, n = 74	*P-*value^a^
Age, years	56 (42–70)	56 (51–60)	57 (53–61)	0.92
Female sex	34 (26)	10 (18)	24 (32)	0.07
CCI score	2.0 (1.0–4.0)	2.0 (0.0–4.0)	2.0 (1.0–4.0)	0.52
Preexisting illness				
Coronary artery disease	21 (16.0)	12 (21.1)	9 (12.2)	0.23
Congestive heart disease	9 (6.9)	6 (10.5)	3 (4.1)	0.18
Hypertension	47 (35.9)	20 (35.1)	27 (36.5)	0.87
Diabetes mellitus	39 (29.8)	17 (29.8)	22 (29.7)	0.99
Pulmonary disease	7 (5.3)	1 (1.8)	6 (8.1)	0.14
Renal disease	17 (13.0)	7 (12.3)	10 (13.5)	0.84
Liver cirrhosis	4 (3.1)	1 (1.8)	3 (4.1)	0.63
Malignancy	6 (4.6)	3 (5.3)	3 (4.1)	0.74
Cardiac arrest characteristics				
Witnessed arrest	80 (61.1)	49 (86.0)	31 (41.9)	< 0.001
Bystander CPR	92 (70.2)	47 (82.5)	45 (60.8)	0.007
Shockable rhythm	45 (34.4)	36 (63.2)	9 (12.2)	< 0.001
Cardiac aetiology	52 (39.7)	36 (63.2)	16 (21.6)	< 0.001
No-flow time, min	11.9 (0.0–13.0)	2.6 (1.1–4.2)	19.0 (8.2–29.8)	< 0.001
Low-flow time, min	22.7 (10.0–31.0)	13.3 (11.1–15.4)	30.0 (26.8–33.1)	< 0.001
Target temperature				0.01
33 ℃	111 (84.7)	43 (75.4)	68 (91.9)	
36 ℃	20 (15.3)	14 (24.6)	6 (8.1)	
Time from ROSC to scan, h				
Magnetic resonance imaging	2.9 (2.0–3.9)	3.1 (2.6–3.6)	3.2 (2.9–3.6)	0.33
Computed tomography	1.2 (1.1–1.3)	1.2 (0.7–1.8)	1.5 (0.9–2.5)	0.13
Systemic hypoxic injury severity				
Lactic acid, mmol/L	4.0 (3.4–4.6)	2.4 (1.9–2.9)	5.1 (4.2–6.0)	< 0.001
Predictors of neurological outcome				
Presence of pupillary light reflex	78 (60)	50 (88)	28 (38)	< 0.001
Serum NSE, ng/ml	32.4 (21.8–52.2)	23.8 (18.5–30.0)	44.0 (29.7–75.1)	< 0.001
Grey-to-white matter ratio	1.23 (1.18–1.30)	1.27 (1.21–1.31)	1.21 (1.16–1.27)	< 0.001

Continuous and categorical variables are presented as median (interquartile range) and number (%), respectively

*CCI* Charlson comorbidity index, *CPR* cardiopulmonary resuscitation, *IQR* interquartile range, *ROSC* return of spontaneous circulation, *NSE* neuron-specific enolase

^a^*P* values are based on *χ*^2^ test for categorical variables and Mann–Whitney U test for continuous variables

^b^Number of patients included in the analysis

### Analysis of ADC values according to outcome groups

**Fig. 3:**
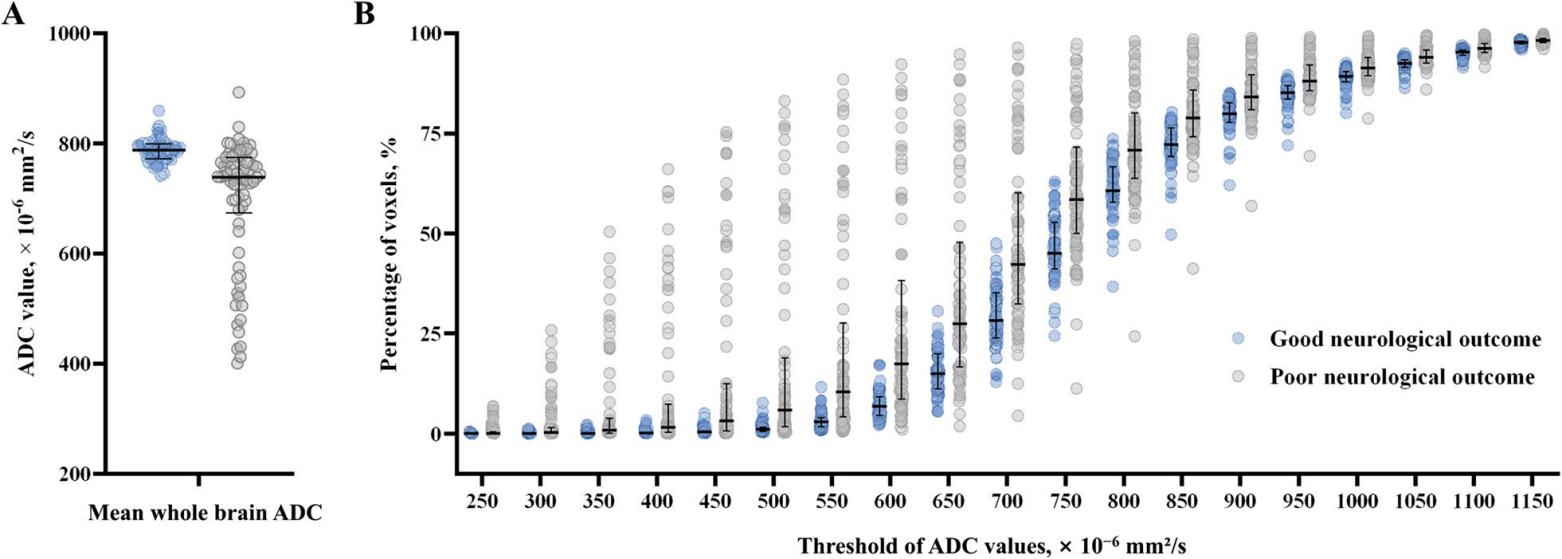
Association between quantitative values of the ADC and the neurological outcomes. Scatter plot and median value (horizontal lines) with interquartile range (error bars) for the association between the quantitative values of the ADC, **A** mean whole brain ADC value and (**B**) the percentage of voxels (y-axis) with ADC below a specific threshold (x-axis), and the neurological outcomes (good, blue circle vs. poor, grey circle). ADC, apparent diffusion coefficient

Figure 3 shows the association between the mean whole brain ADC and ADC-PercentValue of each threshold and neurological outcome. The mean whole brain ADC value in the good neurological outcome group was higher than that in the poor neurological outcome group (787.8 [IQR 771.9–799.6] vs. 739.1 [IQR 673.2–775.1] × 10^−6^ mm^2^/s, *P* < 0.001; Fig. 3A and see Additional file 1, Table S1). In all ranges of ADC values (250 to 1150 × 10^−6^ mm^2^/s), the ADC-PercentValues in the good neurological outcome group were lower than that in the poor neurological outcome group (all *P* < 0.001; Fig. 3B and see Additional file 1: Table S1).

### Analysis of ADC values to predict poor neurological outcomes

Table 2 and Additional file 1: Table S2 show the prognostic performance of the GWR, mean whole brain ADC, and ADC-PercentValue of all range thresholds for poor neurological outcomes 6 months after the ROSC. According to the ROC analysis, the GWR value showed poor prognostic performance (AUC, 0.69; 95% CI 0.60–0.77), while the mean whole brain ADC value and ADC-PercentValue between 350 and 700 × 10^−6^ mm^2^/s presented a good prognostic performance. The other thresholds of ADC-PercentValue showed fair prognostic performance. Furthermore, the mean whole brain ADC value and ADC-PercentValue between 350 and 650 × 10^−6^ mm^2^/s showed significantly better prognostic performance than that of GWR (DeLong, all *P* < 0.05). The mean whole brain ADC value (AUC, 0.83; 95% CI, 0.76–0.89; cut-off value < 739.2 × 10^−6^ mm^2^/s) was a good predictor of poor neurological outcomes, followed by a ADC-PercentValue 450 × 10^−6^ mm^2^/s (AUC, 0.82; 95% CI, 0.74–0.88; cut-off value > 5%) and a ADC-PercentValue 500 × 10^−6^ mm^2^/s (AUC, 0.82; 95% CI, 0.74–0.88; cut-off value > 8%). However, an ADC-PercentValue 600 × 10^−6^ mm^2^/s showed the highest sensitivity (51% [95% CI 39.4–63.0], cut-off value > 17%, Table 2) when the specificity was 100% among the 50 steps of the ADC value.

**Table 2:** Prognostic performance of the voxel-based quantitatively analysed parameters of ADC for the poor neurological outcome and comparison with GWR

Parameters	Cut-off value	AUC (95% CI)	Sensitivity (95% CI)	Specificity (95% CI)	PPV (95% CI )	NPV (95% CI)	TP	FP	TN	FN	*P* value^a^
*CT*											
GWR	<1.11	0.69 (0.60–0.77)	18 (10–28)	100 (94–100)	100 (75–100)	48 (39–58)	12	0	57	62	reference
*DW-MRI*											
Mean whole brain ADC value × 10^−6^ mm²/s	<739.2	0.83 (0.76–0.89)	51 (39–63)	100 (94–100)	100 (91–100)	61 (51–71)	37	0	57	37	0.01
ADC-PercentValue threshold, %											
*Threshold of ADC, × 10*^*−6*^* mm²/s*											
350	>2.2	0.80 (0.73–0.87)	31 (21–43)	100 (94–100)	100 (85–100)	53 (43–63)	23	0	57	51	0.04
400	>3.4	0.81 (0.73–0.87)	34 (23–46)	100 (94–100)	100 (86–100)	54 (44–64)	25	0	57	49	0.03
450	>5.2	0.82 (0.74–0.88)	38 (27–50)	100 (94–100)	100 (88–100)	55 (45–65)	27	0	57	47	0.02
500	>7.8	0.82 (0.74–0.88)	41 (29–53)	100 (94–100)	100 (88–100)	56 (46–66)	30	0	57	44	0.02
550	>11.7	0.81 (0.74–0.88)	43 (32–55)	100 (94–100)	100 (89–100)	58 (47–68)	32	0	57	42	0.03
600	>17.2	0.81 (0.74–0.88)	51 (39–63)	100 (94–100)	100 (91–100)	61 (51–71)	38	0	57	36	0.03

^a^*P* values are based on the DeLong test for comparison of the area under the receiver operating characteristic curve

*ADC* apparent diffusion coefficient, *GWR* grey/white matter ratio, *AUC* area under the receiver operating characteristic curve, *PPV* positive predictive value, *NPV* negative predictive value, *TP* true positive, *FP* false positive, *TN* true negative, *FN* false negative, *CI* confidence interval, *CT* computed tomography, *DW-MRI* diffusion-weighted magnetic resonance image, *ADC-PercentValue threshold* percentage of voxel with ADC value below threshold

**Fig. 4:**
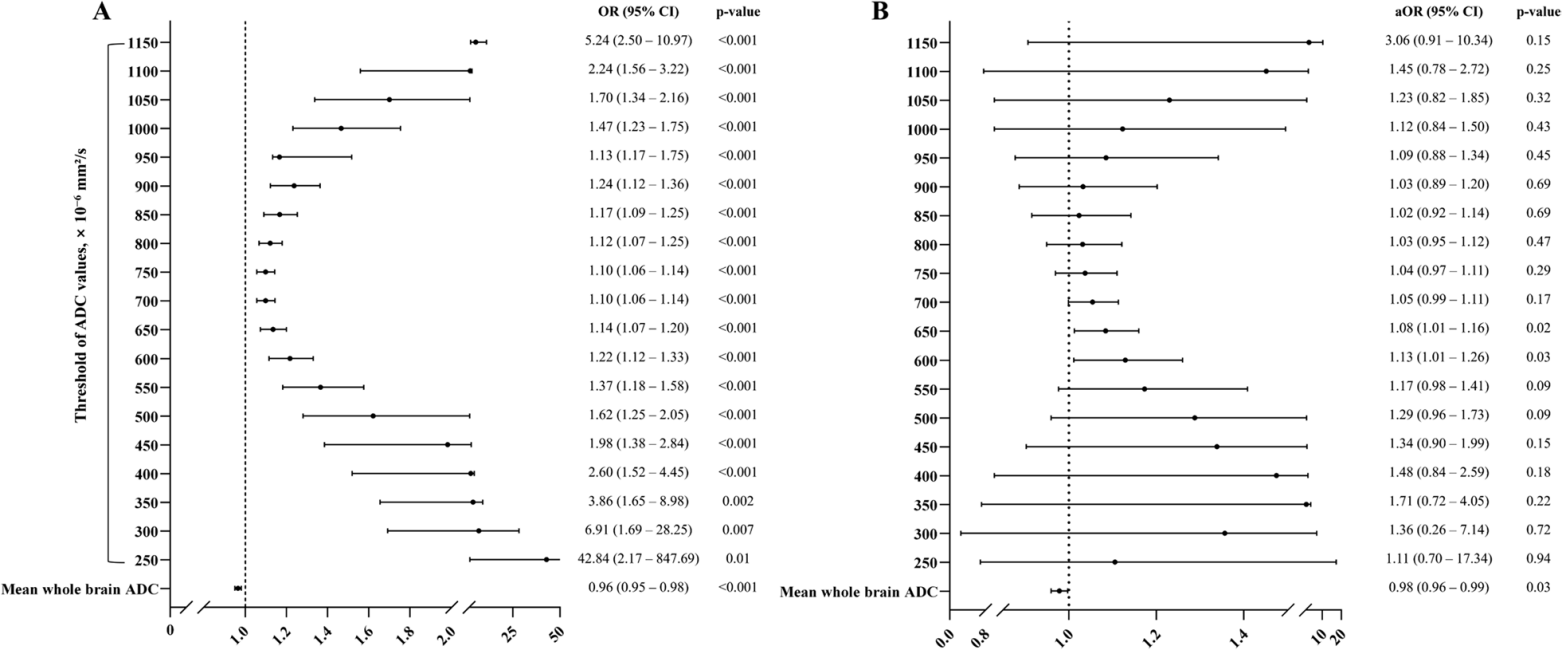
Association between the voxel-based quantitatively analysed parameters of ADC (the mean whole brain ADC and percentage of voxels with ADC below a specific threshold [200 to 1150 × 10^−6^ mm^2^/s]) and the poor neurological outcomes. **A** All parameters are associated with the poor neurological outcome in the univariable analyses. **B** After adjustment with the cardiac arrest characteristics (no- and low-flow time, bystander CPR, witnessed arrest, shockable rhythm, and cardiac aetiology) and serum lactic acid level measured after ROSC, the mean whole brain ADC and the percentage of voxels with ADC value below 600 and 650 × 10^−6^ mm^2^/s were independently associated with the poor neurological outcome. ADC, apparent diffusion coefficient; OR, odds ratio; aOR, adjusted odds ratio

Multivariable analysis revealed that factors such as no-flow time (OR 1.11; 95% CI 1.05–1.17), low-flow time (OR 1.14; 95% CI 1.09–1.19), shockable rhythm (OR 0.09; 95% CI 0.04–0.20), cardiac aetiology (OR 0.16; 95% CI 0.07–0.34), witnessed arrest (OR 0.16; 95% CI 0.05–0.28), bystander CPR (OR 0.32; 95% CI 0.14–0.74), and lactic acid levels (OR 0.32; 95% CI 0.14–0.74) were associated with poor neurological outcomes. Hence, those factors were selected as covariates. The final adjusted model revealed that the mean whole brain ADC (adjusted OR [aOR] 0.98; 95% CI 0.96–0.99, *P* = 0.03), ADC-PercentValues of 600 × 10^−6^ mm^2^/s (aOR 1.13; 95% CI 1.01–1.26, *P* = 0.03), and 650 × 10^−6^ mm^2^/s (aOR 1.08, 95% CI 1.01–1.16, *P* = 0.02) were associated with poor neurological outcomes (Fig. 4).

### Multivariable prediction model for poor neurological outcome

**Fig. 5:**
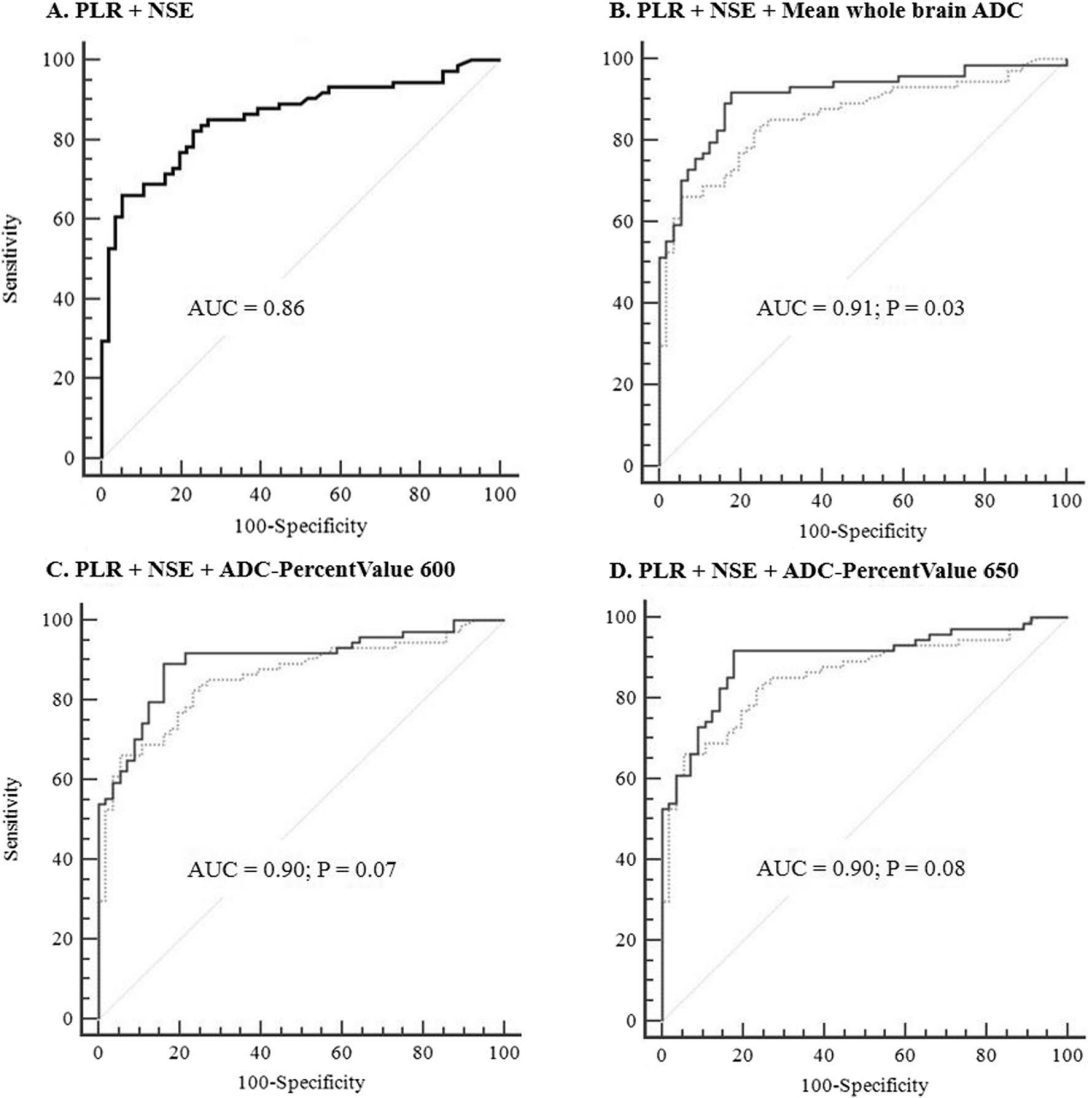
Receiver operating characteristic (ROC) curves of the four models after backward logistic regression. Model B showed improved accuracy (difference between areas, 0.05; *P* = 0.03) and sensitivity compared to the combination of PLR and serum NSE levels. **A** Area under the ROC curve (AUC) 0.86 (95% confidence interval [CI] 0.79–0.92); 30% sensitivity for 100% specificity. **B** AUC 0.91 (95% CI 0.86–0.96); 51% sensitivity and 100% specificity. **C** AUC 0.90 (95% CI 0.85–0.95); 54% sensitivity for 100% specificity. **D** AUC 0.90 (95% CI 0.85–0.95); 53% sensitivity and 100% specificity. PLR, pupillary light reflex; NSE, neuron-specific enolase; ADC, apparent diffusion coefficient; mean whole brain ADC = the average ADC value of the entire brain volume; ADC-PercentValue 600 = the percentage of voxel with ADC values below 600 × 10^−6^mm^2^/s among the entire brain volume; ADC-PercentValue 650 = the percentage of voxel with ADC values below 650 × 10^−6^mm^2^/s among the entire brain volume

Excluding one patient without serum NSE data, we performed a multivariable analysis in 130 patients. We constructed four models to predict poor neurological outcomes using the following: the mean whole brain ADC value, ADC-PercentValues of 600 and 650 × 10^−6^ mm^2^/s, along with PLR and serum NSE levels. By combining information on the absence of PLR, serum NSE levels, and mean whole brain ADC value, a poor neurological outcome was predicted with the highest accuracy (AUC, 0.91; 95% CI, 0.86–0.96) and sensitivity of 51% (95% CI 39–63) at an FPR of 0% (Fig. 5). This model showed improved accuracy (difference between areas, 0.05; *P* = 0.03) and sensitivity of 30% (95% CI 20–42) compared to the combination of PLR and serum NSE levels (AUC, 0.86; 95% CI 0.79–0.92) (Fig. 5).

## Discussion

In this retrospective analysis, the group with poor neurological outcomes had lower mean whole brain ADC values and higher ADC-PercentValue thresholds in all ranges (250 to 1150 × 10^−6^ mm^2^/s). The mean whole brain ADC value and ADC-PercentValue between 350 and 650 × 10^−6^ mm^2^/s showed better prognostic performance than the GWR value. Of these, the mean whole brain ADC value was the best predictor (AUC 0.83; sensitivity 51% when FPR was 0%) of poor neurological outcomes. Multivariable regression analysis of cardiac arrest characteristics showed that mean whole brain ADC values and the ADC-PercentValues 600 and 650 × 10^−6^ mm^2^/s were independently associated with poor neurological outcomes. In predicting neurological outcomes, the multimodal assessment that combined the absence of PLR, serum NSE levels, and mean whole brain ADC value had the highest accuracy (0.91) and sensitivity (51%), at an FPR of 0%, in predicting poor neurological outcomes (CPC 3–5) 6 months after OHCA.

International guidelines for post-cardiac arrest care recommend predicting prognosis 72 h after ROSC to prevent inappropriate WLST in patients with good neurological outcomes [5, 27]. However, the recent COVID-19 pandemic sparked significant discussion regarding the appropriate distribution of medical resources, and several research findings have been reported on the utility of early neurological outcome prediction (i.e. within 6 h after ROSC) in survivors of cardiac arrest [7, 11, 28]. Particularly, early neurological outcome prediction could be helpful when there is a reluctance to provide therapeutic interventions to patients unlikely to benefit, or when treating a patient with an intervention lasting at least 72 h seems unwarranted due to minimal chances of recovery. Nevertheless, despite the potential advantages of early prognostication and our significant findings in this study, it remains insufficient to justify making a clinical decision (i.e. WLST) based entirely on early neurological outcome prediction. It would be pertinent to justify early decision-making for the appropriate allocation of medical resources through early neurological outcome prediction only after confirmatory prospective multicentre evaluation in an unbiased and reproducible setting and after renewed ethical consideration. Therefore, an important ethical consideration in the early prediction of prognosis was to allocate treatment opportunities, such as intensive care units, to survivors of cardiac arrest who were predicted to have good neurological outcomes, rather than making early decisions for withdrawal of life-sustaining treatment (WLST).

In this study, CT-based GWR values obtained within 6 h after the ROSC mostly had poor prognostic prediction performance compared to MRI-based ADC metrics. In the case of CT, observational studies in severe HIBI patients have shown that GWR gradually decreases over time, resulting in poor predictive performance in early CT scans (within 6 h after ROSC) (AUC 0.70), which improved in late CT scans (after > 24 h of ROSC) (AUC 0.80) [29]. Therefore, they reported that CT scans performed after 24 h of ROSC remain an important option in the multimodal approach to neuroprognostication. In contrast, cytotoxic oedema due to HIBI after acute cardiac arrest displays high-signal intensity (HSI) with low ADC much earlier (within 6 h after ROSC) in DW-MRI [1, 2, 5, 13]. Prognostic studies using the presence of HSI on DW-MRI have shown varying sensitivity and specificity, probably due to inconsistency between studies or the lack of a precise definition of HSI [7–9, 18, 19]. Although analysed by the same scanner and neuroradiologist as in our previous study, studies using DW-MRI performed within 6 h and 72 to 96 h after ROSC demonstrated differences in prognostic performance and sensitivity between 0.87 versus 0.97 and 74% versus 93%, respectively [7, 8]. However, the ADC enables quantitative measurement of diffusion changes in brain MRI, with low ADC values indicating restricted diffusion. Yet, there is no consensus on the ideal technique for evaluating the decrease in ADC value in the brain after HIBI [20–23].

When the number of voxels with an ADC value of less than 650 × 10^−6^ mm^2^/s exceeded 10% of the total voxels in the brain, it was associated with poor neurological outcomes, with a specificity of 91% and sensitivity of 72% in predicting those outcomes [20, 21]. Furthermore, a prospective cohort study conducted to validate the threshold of this research, reported a predictive value of 0.79 when the specificity was 96% and sensitivity was 63% [22]. Contrarily, another study did not confirm the prediction of poor neurological outcomes when applying the same threshold criteria (AUC 0.59) [23]. However, they reported that the proportion of brain voxels below 650 × 10^−6^ mm^2^/s, which is required to predict a poor neurological outcome with 100% specificity, was similar at 23.4% compared to 22% in the study by Hirsch et al. [20].

Additionally, a systematic review reported that all studies identified ADC thresholds for the FPR of 0%, often with a sensitivity greater than 50%, including the mean global or regional ADC value of the brain, the proportion of voxels with low ADC value, and a maximum size of MRI clusters with minimum ADC [1, 30]. In the present study, the mean whole brain ADC value demonstrated the best predictive performance (AUC 0.83; sensitivity 51%; specificity 100%) for predicting poor neurological outcomes. Moreover, the proportion of brain voxels with ADC values less than 650 × 10^−6^ mm^2^/s was 30.6%, which was higher than that in previous studies [20, 23]. There are several possible explanations for this discrepancy in the prognostic performance of the quantitative analysis of ADC between studies: differences in (1) the timing of scans after cardiac arrest, (2) the performance of the MRI machine (i.e. the strength of the magnet), and (3) a potential for confounding effects on brain image patterns emerging from neurological disorders other than HIBI after cardiac arrest.

The study by Wouters et al. [23] assumed that the reasons for the differences in results between studies included the differences in the timing of defining neurological outcomes after cardiac arrest, the timing of MRI scans, the MRI analysis method, and the definition of poor neurological outcomes. Furthermore, changes in the ADC can be apparent in the early post-arrest period. However, in many patients, changes in the ADC are not apparent until more than 24 h post-arrest [17]. In this study (within 6 h after ROSC), the DW-MRI was performed earlier compared to other studies (2–7 d after ROSC) [20–23]. Therefore, it is estimated that a higher proportion of brain voxels with an ADC value of less than 650 × 10^−6^ mm^2^/s is required to predict a poor neurological prognosis when the FPR is 0%, compared to previous studies, owing to the time-dependent occurrence of brain oedema (cytotoxic oedema) after HIBI [20–23]. In our previous study, ADC-PercentValue of 520 × 10^−6^ mm^2^/s obtained between 72 and 96 h after ROSC demonstrated a sensitivity of 73.3% (95% CI 44.9–92.2) at a cut-off value > 4.9% when the FPR was 0% [8]. However, in the present study, with the most similar ADC threshold at 500 × 10^−6^ mm^2^/s, the cut-off value exceeded 8% (sensitivity 41%; 95% CI 29–53). In addition to the DW-MRI, the GWR measured on brain CT also showed differential prognostic performance according to the timing of the scan in relation to cardiac arrest. Therefore, we suggest that it would be appropriate to adjust the cut-off threshold of FPR 0% at specific ADC thresholds based on the MRI scan time point.

Additionally, previous studies have compared images obtained from 1.5 T and 3 T MRI scanners by integrating them without distinguishing between them. However, we are concerned whether the 1.5 T and 3 T MRI scanners, due to their different imaging protocols, yield the same ADC thresholds to represent the size of restricted diffusion in HIBI. Furthermore, it is established that 3 T provides much better contrast, resolution and signal-to-noise ratio in DWI compared to 1.5 T MRI scanners [31, 32]. Although we were unable to find comparative data for HIBI, our concern is supported by the findings of Tang et al. who examined the use of DWI for the quantitative analysis of focal liver lesions and found that the ADC thresholds for both the largest solid area and the maximum diameter of the lesion differed between the 1.5 T and 3 T protocols [33].

To the best of our knowledge, none of the studies for the quantitative analysis of DW-MRI considered the confounding effects on the brain image patterns arising due to non-HIBI conditions after cardiac arrest, such as a metabolic crisis (e.g. hypoglycaemia, hyperammonaemia, or other conditions), seizures, or opioid intoxication [34]. In particular, seizures occur in 15–44% of the patients with HIBI after cardiac arrest [35, 36]. In view of the different manifestation in DW-MRI and the high prevalence of such medical conditions, the potential impact of non-HIBI conditions on the brain image patterns in DW-MRI needs to be considered in the future studies.

International guidelines for post-cardiac arrest care recommend a multimodal approach to predict prognosis [5]. However, it is not always possible to obtain all the predictive variables desired at the time of prognosis prediction [3]. Therefore, in this study, we used only a combination of easily available clinical variables, such as PLR and serum NSE levels within 6 h after ROSC, instead of identifying the optimal timing for each predictor to achieve the best predictive performance. Furthermore, considering that serum NSE levels and neuroimaging variables are minimally influenced by sedatives and neuromuscular blockers administered to patients, they are useful in this context [1–3, 5]. The predictive performance of the combination of the absence of PLR, serum NSE levels and mean whole brain ADC value was excellent (AUC 0.91), with a sensitivity of 51% at an FPR of 0%. Although the sensitivity was slightly lower than the 60% sensitivity at an FPR of 0% reported in an external validation study of the 2020 ERC/ESICM prognostic algorithm [35], it still holds considerable value when considering the prediction timing (within 6 h after ROSC vs. 72 to 96 h after ROSC). Additionally, in a recent study predicting a poor neurological outcome based on the presence or absence of HSI in DW-MRI within 6 h and up to 10 days after the ROSC, the sensitivity values at 0% FPR were 74.2% and 89.6%, respectively [7, 19]. Generally, these values are higher than those reported in previous studies using voxel-based quantitative ADC analysis [20–23]. Therefore, varying results obtained due to differences in analytical methods suggest the need for a prospective comparative study using MRI conducted at a more consistent time, considering inter-rater agreements and the applicability to clinical settings.

### Limitations

This study has several limitations. First, it focused solely on patients who underwent DW-MRI within 6 h after the ROSC in a single centre, resulting in a small sample size. Most researchers are reluctant to obtain MRI scans from patients who are intubated and critically ill. No adverse events or complications related to early MRI were observed during the study period. Therefore, prospective multicentre studies are required to generalise these results. Second, due to the lack of universal consensus on the ideal technique for MRI analysis, the results can vary depending on the approach used. Third, when predicting outcomes in survivors of cardiac arrest at an early stage (i.e. within 6 h after the ROSC), it is preferable to focus on good neurological outcomes. However, in this study, our objective was to predict poor neurological outcomes. This is because there are many studies on the prediction of poor neurological outcomes in general and there are limitations in understanding values based on the results of single-centre studies with small sample sizes. However, Sandroni et al. reported in their study that the accuracy of predicting good neurological outcomes was inversely proportional to the accuracy of predicting poor neurological outcomes [3]. In other words, the specificity for predicting good neurological outcomes is equivalent to the sensitivity for predicting poor neurological outcomes and vice versa. Finally, MRI analysis using FSL software was performed blind to the clinical outcomes of the patients after their medical care was completed. However, the qualitative findings of early DW-MRI (presence or absence of HSI) were exposed to clinicians, potentially introducing a self-fulfilling prophecy bias. However, our institution did not allow WLST during TTM, unless the patient was diagnosed as brain dead; in this study, WLST during TTM did not occur, although some patients were pronounced dead according to circulatory or neurological criteria despite maximal support.

## Conclusion

In this study, early (within 6 h after the ROSC) voxel-based quantitative ADC analysis in survivors of OHCA using a 3 T MRI scanner showed that mean whole brain ADC values and ADC-PercentValues 600 and 650 × 10^−6^ mm^2^/s were significantly associated with poor neurological outcomes 6 months after OHCA. Among the various ADC analyses, the mean whole brain ADC value of the brain exhibited the highest accuracy regarding predictive performance and sensitivity at an FPR of 0%. Furthermore, the addition of the mean whole brain ADC value to the combination of the absence of PLR and serum NSE levels significantly improved the accuracy of predicting poor neurological outcomes in survivors of OHCA. Future multicentre studies with a larger sample size and consensus on the ideal technique for MRI analysis are warranted.

### Supplementary Information

**Additional file 1**. **1. Table S1**: Associations between the quantitatively analyzed predictors of DW-MRI and neurological outcome. **2. Table S2**: Prognostic performance of the voxel-based quantitatively analyzed parameters of ADC for the poor neurological outcome and comparison with GWR.

## Data Availability

The datasets used and/or analysed during the current study are available from the corresponding author on reasonable request.

## Abbreviation

ADC: Apparent diffusion coefficient
aOR: Adjusted odds ratio
AUC: Area under the receiver operating characteristic curve
CC: Corpus callosum
CCI: Charlson comorbidity index score
CI: Confidence interval
CN: Caudate nucleus
CNUH: Chungnam National University Hospital
CPC: Cerebral performance category
CPR: Cardiopulmonary resuscitation
CT: Computed tomography
DW-MRI: Diffusion-weighted magnetic resonance image
ECMO: Extracorporeal membrane oxygenation
FPR: False positive rate
FSL: FMRIB Software Library
GWR: Grey/white matter ratio
HIBI: Hypoxic ischaemic brain injury
HSI: High-signal intensity
IQR: Interquartile range
MRI: Magnetic resonance imaging
NSE: Neuron-specific enolase
OHCA: Out-of-hospital cardiac arrest
OR: Odds ratio
P: Putamen
PIC: Posterior limb of the internal capsule
PLR: Pupillary light reflex
ROC: Receiver operating characteristic
ROSC: Return of spontaneous circulation
TTM: Target temperature management
WLST: Withdrawal of life-sustaining treatment
ADC-PercentValue threshold: Percentage of voxels with ADC values below thresholds
