# Supplementary material for: Quantitative analysis of early apparent diffusion coefficient values from MRIs for predicting neurological prognosis in survivors of out-of-hospital cardiac arrest: an observational study
Source: Crit Care. 2023 Oct 25;27:407. doi: 10.1186/s13054-023-04696-z (PMC10599006; doi:10.1186/s13054-023-04696-z)
Supplement: Supplementary file 1 — Additional file 1. 1. Table S1: Associations between the quantitatively analyzed predictors of DW-MRI and neurological outcome. 2. Table S2: Prognostic performance of the voxel-based quantitatively analyzed parameters of ADC for the poor neurological outcome and comparison with GWR. [file 13054_2023_4696_MOESM1_ESM.docx]

**Supplemental materials**

**Title: Quantitative analysis of early apparent diffusion coefficient values from MRIs for predicting neurological prognosis in survivors of out-of-hospital cardiac arrest: an observational study**

**eAppendix 1. Table S1.** Associations between the quantitatively analyzed predictors of DW-MRI and neurological outcome

**eAppendix 2. Table S2.** Prognostic performance of the voxel-based quantitatively analyzed parameters of ADC for the poor neurological outcome and comparison with GWR

**1. Table S1.** **Associations between the voxel-based quantitatively analyzed parameters of ADC and neurological outcome**

| Parameters | | Overall cohort, n = 131 | Good neurological outcome, n = 57 | Poor neurological outcome, n = 74 | *P*-value^a^ |
| --- | --- | --- | --- | --- | --- |
| Mean whole brain ADC value ^b^, × 10^−6^ mm^2^/s | | 769.7 (733.3–792.1) | 787.8 (771.9–799.6) | 739.1 (673.2–775.1) | <0.001 |
| ADC-PercentValue thresholds, % | |  | | | |
| Thresholds  of ADC,  × 10^−6^ mm²/s | 250 | 0.03 (0.02–0.22) | 0.02 (0.01–0.04) | 0.10 (0.03–0.50) | <0.001 |
|  | 300 | 0.09 (0.04–0.54) | 0.05 (0.03–0.10) | 0.29 (0.08–1.55) | <0.001 |
|  | 350 | 0.19 (0.09–1.23) | 0.11 (0.07–0.18) | 0.83 (0.16–3.80) | <0.001 |
|  | 400 | 0.36 (0.18–2.61) | 0.19 (0.15–0.36) | 1.60 (0.31–7.37) | <0.001 |
|  | 450 | 0.84 (0.39–4.74) | 0.46 (0.31–0.72) | 3.18 (0.72–12.47) | <0.001 |
|  | 500 | 2.02 (0.96–7.33) | 1.12 (0.65–1.63) | 5.91 (1.78–18.86) | <0.001 |
|  | 550 | 4.40 (2.46–11.63) | 2.93 (1.71–3.97) | 10.47 (4.18–27.65) | <0.001 |
|  | 600 | 9.85 (5.62–19.89) | 6.86 (4.60–9.23) | 17.43 (8.66–38.24) | <0.001 |
|  | 650 | 21.00 (13.47–30.61) | 14.94 (11.20–19.90) | 27.44 (16.72–47.79) | <0.001 |
|  | 700 | 35.52 (27.00–45.86) | 28.32 (23.89–35.17) | 42.26 (32.41–60.16) | <0.001 |
|  | 750 | 52.05 (44.19–62.22) | 45.03 (41.14–52.80) | 58.40 (49.99–71.64) | <0.001 |
|  | 800 | 66.31 (59.79–72.36) | 60.63 (57.73–66.63) | 70.88 (63.81–80.05) | <0.001 |
|  | 850 | 75.87 (71.33–79.58) | 72.28 (69.27–76.40) | 78.82 (74.14–85.77) | <0.001 |
|  | 900 | 82.14 (79.33–85.08) | 79.86 (77.74–82.67) | 84.13 (80.90–89.64) | <0.001 |
|  | 950 | 86.56 (84.62–89.28) | 85.14 (83.63–86.90) | 88.04 (85.72–92.06) | <0.001 |
|  | 1000 | 90.22 (88.74–91.98) | 89.25 (87.92–90.50) | 91.27 (89.35–93.95) | <0.001 |
|  | 1050 | 93.05 (92.07–94.40) | 92.49 (91.49–93.38) | 93.99 (92.56–95.78) | <0.001 |
|  | 1100 | 95.61 (94.96–96.45) | 95.27 (94.48–95.82) | 96.24 (95.25–97.40) | <0.001 |
|  | 1150 | 97.91 (97.56–98.34) | 97.76 (97.32–98.00) | 98.23 (97.72–98.74) | <0.001 |

Data are presented as median value and interquartile range.

^a^, P values are based on Mann-Whitney U test for continuous variables.

^b^, Mean whole brain ADC value is defined as the average ADC of the entire brain volume and presented as median value and interquartile range .

^c^, The percentages of voxels with ADC below thresholds was calculated based on the total voxel numbers of ADC values between 200 and 2000 × 10^−6^ mm^2^/s.

**Abbreviations**: ADC, apparent diffusion coefficient; IQR, interquartile range

**2. Table S2**. Prognostic performance of the voxel-based quantitatively analyzed parameters of ADC for the poor neurological outcome and comparison with GWR

| Parameters | | Cut-off value | AUC  (95% CI) | Sensitivity  (95% CI) | Specificity  (95% CI) | PPV  (95% CI ) | NPV  (95% CI) | TP | FP | TN | FN | P value ^a^ |
| --- | --- | --- | --- | --- | --- | --- | --- | --- | --- | --- | --- | --- |
| CT | | | | | | | | | | | | |
| GWR | | <1.11 | 0.69 (0.60–0.77) | 18 (10–28) | 100 (94–100) | 100 (75–100) | 48 (39–58) | 12 | 0 | 57 | 62 | reference |
| DW-MRI | | | | | | | | | | | | |
| ADC-PercentValue thresholds, % | |  | | | | | | | | | | |
| Thresholds  of ADC,  × 10^−6^ mm²/s | 250 | >0.6 | 0.76 (0.68–0.83) | 23 (14–34) | 100 (94–100) | 100 (81–100) | 50 (41–60) | 17 | 0 | 57 | 57 | 0.21 |
|  | 300 | >1.3 | 0.78 (0.70–0.85) | 28 (19–40) | 100 (94–100) | 100 (84–100) | 52 (42–61) | 21 | 0 | 57 | 53 | 0.10 |
|  | 700 | >47.6 | 0.80 (0.72–0.87) | 39 (28–51) | 100 (94–100) | 100 (88–100) | 56 (46–66) | 29 | 0 | 57 | 45 | 0.05 |
|  | 750 | >63.0 | 0.79 (0.71–0.86) | 39 (28–51) | 100 (94–100) | 100 (88–100) | 56 (46–66) | 29 | 0 | 57 | 45 | 0.07 |
|  | 800 | >73.6 | 0.79 (0.71–0.86) | 38 (27–50) | 100 (94–100) | 100 (88–100) | 55 (45–65) | 28 | 0 | 57 | 46 | 0.07 |
|  | 850 | >80.3 | 0.79 (0.71–0.85) | 41 (29–53) | 100 (94–100) | 100 (88–100) | 56 (46–66) | 30 | 0 | 57 | 44 | 0.09 |
|  | 900 | >85.1 | 0.78 (0.70–0.85) | 43 (32–55) | 100 (94–100) | 100 (89–100) | 58 (47–68) | 32 | 0 | 57 | 42 | 0.10 |
|  | 950 | >89.5 | 0.77 (0.69–0.84) | 39 (28–51) | 100 (94–100) | 100 (88–100) | 56 (46–66) | 29 | 0 | 57 | 45 | 0.15 |
|  | 1000 | >92.6 | 0.77 (0.68–0.84) | 37 (26–49) | 100 (94–100) | 100 (87–100) | 55 (45–65) | 27 | 0 | 57 | 47 | 0.17 |
|  | 1050 | >95.0 | 0.76 (0.67–0.83) | 31 (21–43) | 100 (94–100) | 100 (85–100) | 53 (43–63) | 23 | 0 | 57 | 51 | 0.24 |
|  | 1100 | >97.0 | 0.75 (0.67–0.83) | 31 (21–43) | 100 (94–100) | 100 (94–100) | 53 (43–63) | 22 | 0 | 57 | 52 | 0.25 |
|  | 1150 | >98.6 | 0.75 (0.67–0.83) | 30 (20–42) | 100 (94–100) | 100 (85–100) | 52 (43–62) | 22 | 0 | 57 | 52 | 0.25 |

^a^, P values are based on the DeLong test for comparison of the area under the receiver operating characteristic curve

**Abbreviations**: ADC, apparent diffusion coefficient; GWR, gray-white matter ratio; AUC, area under the receiver operating characteristic curve; PPV, positive predictive value; NPV, negative predictive value; TP, true positive; FP, false positive; TN, true negative; FN, false negative; CI, confidence interval; CT, computed tomography; DW-MRI, diffusion-weighted magnetic resonance image; ADC-PercentValue threshold, percentage of voxel with ADC value below threshold
